# Impact of stem cell marker expression on recurrence of TACE-treated hepatocellular carcinoma post liver transplantation

**DOI:** 10.1186/1471-2407-12-584

**Published:** 2012-12-07

**Authors:** Zhen Zeng, Jinyu Ren, Maura O’Neil, Jie Zhao, Brian Bridges, Josiah Cox, Bashar Abdulkarim, Timothy M Schmitt, Sean C Kumer, Steven A Weinman

**Affiliations:** 1 Beijing 302th Hospital, Beijing, 100039, People’s Republic of China; 2 Department of Internal Medicine, University of Kansas Medical Center, Kansas City, KS 66160, USA; 3 Department of Pathology, University of Kansas Medical Center, Kansas City, KS, 66160, USA; 4 Department of Microbiology, University of Kansas Medical Center, Kansas City, KS, 66160, USA; 5 Department of Surgery, University of Kansas Medical Center, Kansas City, KS, 66160, USA

**Keywords:** Cancer stem cells, EpCAM, CD133, CD90, CD44, Transarterial chemoembolization

## Abstract

**Abstract:**

**Background:**

Liver transplantation is the most effective therapy for cirrhosis-associated hepatocellular carcinoma (HCC) but its utility is limited by post-transplant tumor recurrence. Use of the Milan, size-based criteria, has reduced recurrence rate to less than 10% but many patients remain ineligible. Reduction of tumor size with local therapies has been used to “downstage” patients to allow them to qualify for transplantation, but the optimal criteria to predict tumor recurrence in these latter patients has not been established. The existence of a progenitor cell population, sometimes called cancer stem cells (CSCs), has been proposed to be one mechanism accounting for the chemotherapy resistance and recurrence of hepatocellular carcinoma. The aim of this study was to determine if transcatheter arterial chemoemolization (TACE) treated tumors have increased CSC marker expression and whether these markers could be used to predict tumor recurrence.

**Methods:**

Formalin fixed specimens were obtained from 39 HCC liver explants (23 with no treatment and 16 after TACE). Immunohistochemical staining was performed for EpCAM, CD44, CD90, and CD133. Staining for each marker was scored 0–3 by evaluating the number and intensity of positive tumor cells in 5 hpf of tumor in each specimen.

**Results:**

TACE treated tumors displayed greater necrosis and fibrosis than non-TACE treated samples but there were no differences in morphology between the viable tumor cells of both groups. In TACE treated specimens, the staining of both EpCAM and CD133 was greater than in non-TACE specimens but CD44 and CD90 were the same. In the TACE group, the presence of high EpCAM staining was associated with tumor recurrence. Four of ten EpCAM high patients recurred while 0 of 6 EpCAM low patients recurred (P = 0.040). None of the other markers predicted recurrence.

**Conclusion:**

High pre-transplant EpCAM staining predicted HCC recurrence. This suggests that the abundance of tumor cells with a CSC phenotype may be a critical factor in the likelihood of tumor recurrence in patients receiving liver transplantation after TACE.

## Background

Hepatocellular carcinoma (HCC) is one of the most prevalent cancers in Asia and Africa, and ranks as the third most frequent cause of cancer-related death [1,2]. Its incidence is increasing in the western world due to increased prevalence of hepatitis virus C infection [3,4]. Although considerable advances have been made in the treatment of HCC, particularly through surgical resection, liver transplantation, tumor ablation and chemotherapy, the mortality rate remains high, and tumor recurrence after surgery or transplantation remains a frequent problem. A better understanding of the biology of the tumors and the factors that predict recurrence post surgery would have a major impact on the management of the disease.

For the majority of patients who develop HCC in the setting of cirrhosis, liver transplantation remains the primary surgical approach. While early experience showed a high proportion of patients recurring, current practice of limiting transplantation eligibility to patients with lower tumor volume and absence of vascular invasion, the Milan criteria [5], has decreased recurrence rate to less than 10% at most centers [6]. Unfortunately, there is broad recognition that criteria based solely on tumor size, while helpful, include some patients who will recur and likely exclude some patients from transplantation who would not have recurred. In addition patients frequently receive local therapy to the tumor prior to transplant, and in many cases, tumor shrinkage via therapeutic “downstaging” is required for patients to meet transplant eligibility requirements [7,8].

Successful downstaging has been shown to result in overall transplant outcomes similar to those achieved for patients who start out within Milan criteria [9], however, approximately 15-30% of patients for whom downstaging is attempted progress in spite of treatment and thus are excluded from transplantation [9,10]. The success of downstaging, therefore, is partly a result of the ability of the followup period to exclude patients with aggressive and poorly responsive tumors. The biological behavior of the tumors post-treatment is variable and the concern has been raised that in some cases, TACE itself might select for or induce more aggressive tumors [11]. Whether or not this is relevant to subgroups of patients and whether a better understanding of tumor biology might lead to improved ability to predict post-transplant tumor behavior remains uncertain.

Hepatocellular carcinomas consist of a heterogeneous group of cells that have varying ability to proliferate and seed new tumors [12]. It has been proposed that a sub population of cells variously called tumor initiating cells, cancer progenitor cells, or cancer stem cells (CSCs) serves as a proliferation reservoir, is able to seed new tumors with very low inoculum levels and is responsible for recurrence and metastases. While these cells are not true pluripotent stem cells, they possess characteristics of stem cells in that they can give rise to all the cell types in the tumor. They are relatively chemotherapy resistant and are a strong candidate for the source of intrahepatic HCC recurrence post liver transplantation [13]. There is no general consensus on the best markers to identify these cells and as a result there is no clear consensus of whether they play a major role in HCC. Several prior studies have examined CD133, CD44, CD90, CD13 and EpCAM as possible candidate CSC markers in HCC [14-19].

Several makers, particularly EpCAM and CK19 have been shown to correlate with more aggressive tumor behavior [20,21]. We therefore sought to determine if stem cell marker expression predicted tumor recurrence after transplantation, particularly in patients who had undergone transcatheter arterial chemoembolization (TACE). In the present study, we compared the phenotypic expression of four markers in HCCs resected from patients undergoing liver transplantation either with or without prior TACE treatment of the tumor, and determined whether any of these correlated with the likelihood of tumor recurrence. The results demonstrate that EpCAM and CD133 positive cells were higher in the post-TACE tumors and higher pretransplant EpCAM immunostaining significantly correlated with the risk of 2-year tumor recurrence.

## Methods

### Ethics statement

The research involving human subjects reported in this study involved retrospective analysis of tissue samples and de-identified clinical data from patients who had agreed to allow their explanted tissue samples to be included in a tissue Biorepository. The research was approved by the University of Kansas Medical Center Human Subjects Committee, under approval numbers HSC# 11378 and HSC#12800. All participating subjects gave written informed consent. All clinical investigations were conducted according to the principles expressed in the Declaration of Helsinki.

### Tissue collection

Samples of paraffin-embedded sections of explanted HCC and adjacent liver specimens were obtained from the University of Kansas Cancer Center's (KUCC) Biospecimen Shared Resource or the University of Kansas Liver Center Tissue Bank. These were obtained from 39 patients who underwent liver transplantation at the University of Kansas Medical Center (KUMC) from January, 2004 to July ,2010. Clinical data associated with the specimens was recorded without patient identification and all procedures were approved by the Human Studies Committee at KUMC. Sixteen cases were from patients whose tumor had been previously treated by transcatheter arterial chemoembolization (TACE) using doxorubicin and lipiodol and 23 cases were from patients who did not receive any specific tumor treatment prior to liver transplantation. All post-TACE HCCs examined in this study had microscopic foci of viable carcinoma as well as coagulative necrosis consistent with the effect of therapy. Survival data were determined at the last follow-up period for living patients. For those patients in whom tumor relapse occurred, relapse time was taken to be the interval between the date of liver transplantation and the date of diagnosis of any type of relapse with either intrahepatic recurrence or extrahepatic metastasis defined as the end points.

### Immunohistochemical staining

Paraffin-embedded, formalin-fixed liver tissues were cut into 5-μm sections and placed on polylysine-coated slides. Antigen retrieval was achieved with a steam pressure cooker for 10 min in sodium citrate buffer (10 mM sodium citrate 0.05% Tween 20, pH 6.0). The sections were incubated with 3% hydrogen peroxide/PBS for 20 min and then rinsed twice in washing buffer TBS-T for 5 min each. Slides were then blocked by incubation for 1 h at room temperature with 5% goat serum in TBS-T. After blocking, samples were incubated with primary antibodies (rabbit anti-CD133, cat# ab19898, Abcam, 1:250; anti-EpCAM cat# ab68892, Abcam, 1:1000; anti-CD44, cat# ab65829, Abcam 1:250, anti-CD90, cat# ab92574, Abcam,1:100) in 1% goat serum in TBS-T) and incubated overnight at 4°C. After washing, slides were then incubated with HRP-labeled Polymer (EnVision + System, Dako, Carpinteria, CA) and either anti-rabbit (K4002) or anti-mouse (K4000) secondary antibody, for 1 h at room temperature, and then developed with AEC substrate Chromogen (Dako, K3464) and counterstained by Mayer’s hematoxylin.

**Figure 1:**
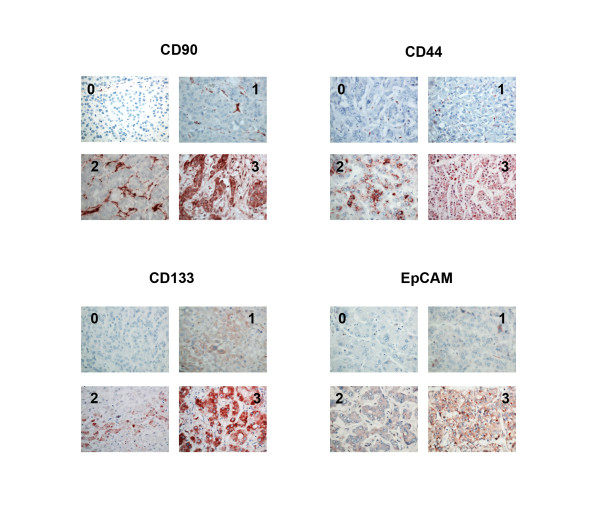
**Pattern and intensity of staining for potential cancer stem cell markers in hepatocellular carcinoma specimens.** Formalin-fixed paraffin-embedded human HCC samples were immunostained for CD90, CD44, CD133, and EpCAM and intensity of staining was assessed as described in methods. For each marker, example images are shown demonstrating the staining pattern for each of the intensity grades.

For the evaluation of CD133, CD90, CD44 and EpCAM staining, three independent investigators examined the slides without related clinical information. The intensity of CD133, CD90, CD44 and EpCAM staining was scored on a four point scale. For CD90 and CD44, staining varied little in intensity and was primarily differentiated by the number of positive cells. Score 0 was less than 25% positive; score 1 was 25%-50% positive; score 2 was 50%-75% positive; and score 3 was higher than 75% of cells positive. For EpCAM and CD133, there was variablity in staining intensity as well as in number of positive cells. For these markers, a composite scale taking into account both number and intensity was used. When high intensity staining was present, the scale was the same as for CD90 and CD44, but when staining of varying intensity was present in most cells, the score was based on overall intensity with score 1 being no staining, score 2 faint staining, score 3 moderate staining and score 4 representing strong staining. The final score was the mean value of scores from three observers. Examples of these are shown in Figure 1. Scores 0 and 1 were considered as low expression, whereas scores 2 and 3 were considered as high expression.

### Statistical analysis

Statistical analysis and graphical presentation were performed using SPSS (v17.0) software for Windows (SPSS Inc., Chicago, IL). The clinicopathological parameters were compared with the Mann–Whitney *U*-test and Wilcoxon test .CD44, CD90, CD133 and EpCAM expression were compared with the *χ*^2^ test according to the immunoreactive score of each tissue section. The Cox regression model was used to perform univariate and multivariate analyses. The recurrence rate was calculated using the Kaplan–Meier method, and the resulting curves were compared by the log-rank test. P < 0.05 was considered significant.

## Results

### Clinico-pathological features

Clinico-pathological characteristics of the post-TACE and non-TACE HCCs are shown in Table 1 and the details of individual patients, including the reasons for the decision whether or not to perform TACE are listed in Tables 2 and 3. In the post-TACE group, the underlying disease etiology was HCV in 13 cases, HBV in 2 cases and cryptogenic cirrhosis in 1 case. In the non-TACE group, 21 cases were due to HCV, 1 case to HBV and one to primary sclerosing cholangitis. These patients reflect a period when transplant wait times were relatively short at the University of Kansas and patients with similar tumor sizes sometimes underwent TACE and other times did not. For the TACE group, 8/16 (50%) patients were outside of Milan and the TACE was performed for downstaging, but the other half of the patients were within Milan criteria and the TACE was performed in an attempt to prevent progression. For the non-TACE group 21/23 (91%) were within Milan criteria and only 2/23 (9%) were outside of Milan criteria but received transplants based on their MELD scores alone. Mean largest tumor size was greater in the TACE group (3.8 vs 2.9 cm) as was AFP, but these differences were not statistically significant (Table 1) Other features such as tumor number, tumor differentiation, gender , age, or TNM stage did not differ between the groups. We attempted to determine pre-transplant tumor growth rate to assess whether the TACE patients had more rapidly growing tumors than non-TACE patients, but this did not appear to be the case. Multiple pre-TACE imaging studies were available in 9/16 TACE patients and demonstrated a mean tumor diameter increase rate of 0.08 ± 0.12 cm/month. Multiple imaging studies were only available in 5/23 non-TACE patients but in those patients growth rates were somewhat greater at 0.16 ± 0.15 cm/month. Due to the limited numbers of patients with multiple scans it is not possible to determine the biological significance of this finding.

**Table 1:** Clinical and pathological features of samples included in the study

	**TACE (n = 16)**	**No-TACE (n = 23)**	**P value**
**Age** (years)	56(48–68)	55(45–68)	0.363
**Gender** (F/M)	2/14	5/18	0.460
**AFP** (ng/ml)	660(3–7245)	382(3–2809)	0.114
**Tumor Num.** (Single/Multiple)	8/8	14/9	.0.365
**Largest Tumor size** Mean (range) (mm)	38 (20–76)	29(3–60)	0.069
**Differentiation**			0.156
Well	1	7	
Moderately	12	14	
Poor	3	2	
**TNM Stage**			0.365
I	8	14	
II	8	9	

**Table 2:** Characteristics of individual patients

**No-TACE**																	
**Patient Number**	**Age**	**Gender**	**Etiology**	**AFP**	**# Tumors at Dx**	**Size (cm) pre-op**	**Within Milan**	**Growth rate (cm/mo.)**	**Largest tumor dia by path**	Days to Transplant*	**Recur**	**Time to Recur**	**Reasons not to TACE**				
													**A**	**B**	**C**	**D**	**E**
N1	62	F	HCV	20	2	1.9, 1.6	Y	N/A	1.6	49	N	N/A	X				
N2	48	M	HCV	13	1	1.9	Y	N/A	2	340	N	N/A	X				
N3	49	F	HCV	1207	3	1.3, 2.0, 2.5	Y	N/A	3.4	188	N	N/A	X				
N4	52	F	HCV	107	2	1.0, 2.1	Y	N/A	2.3	160	N	N/A	X				
N5	65	M	HCV	5.3	2	N/A	N/A	N/A	1.1	12	N	N/A				X	
N6	56	M	HBV	4	1	4.8	Y	N/A	3.5	59	N	N/A	X				
N7	68	M	HCV	1327	2	1.4, 2.6	Y	0.12	2.8	205	N	N/A					X
N8	46	F	HCV	2809	1	3.8	Y	0.2	6	115	N	N/A	X		X		
N9	48	M	HCV	686	1	3	Y	0.4	3.6	116	N	N/A	X				
N10	53	M	HCV	3	1	1.5	Y	0.1	2.5	190	N	N/A	X				
N11	45	M	HCV	4	1	5.4	N	N/A	5.1	45	N	N/A		X			
N12	57	M	HCV	6	1	2.8	Y	N/A	4	41	Y	24mo.	X				
N13	55	M	HCV	11	2	0.9, 1.6	Y	N/A	2.3	38	N	N/A	X				
N14	49	M	HCV	7	1	5.3	N	N/A	5.5	87	N	N/A		X			
N15	53	M	HCV	2461	1	2.8	Y	N/A	5	95	N	N/A	X				
N16	55	M	HCV	7	2	1.0, 0.8	Y	N/A	1	0	N	N/A				X	
N17	52	M	HCV	18	1	2.5	Y	N/A	3.1	128	N	N/A	X		X		
N18	56	F	HCV	19	1	3.3	Y	N/A	3.5	117	N	N/A	X		X		
N19	68	M	HCV	9	2	2.2, 1.6	Y	0	2	377	N	N/A	X		X		
N20	56	M	HCV	7	1	N/A	N/A	N/A	1	0	N	N/A				X	
N21	53	M	PSC	40	2	N/A	N/A	N/A	0.3	0	N	N/A				X	
N22	64	M	HCV	20	1	2.1	Y	N/A	3.5	179	N	N/A	X				
N23	49	M	HCV	3.6	1	N/A	N/A	N/A	1.5	0	N	N/A				X	

A = Patient was within Milan.

B = Outside Milan but eligible for transplant by MELD with a short waiting time expected.

C = Patient could not tolerate procedure.

D = Tumor not appreciated pre-op.

E = Procedure planned, not able to perform prior to transplant.

* = Days from HCC diagnosis to transplant.

**Table 3:** Characteristics of individual patients

**TACE**														
**Patient Number**	**Age**	**Gender**	**Etiology**	**AFP**	**# Tumors at Dx**	**Size (cm) pre-op**	**Within Milan**	**Pre-TACE Growth rate (cm/mo.)**	**Largest tumor dia by path**	Days to Transplant*	**Recur**	**Time to Recur**	**Reasons for TACE**	
													**A**	**B**
T1	61	M	Cryptogenic	7254	1	6.3	N	N/A	5	241	N		X	
T2	50	M	HCV	27.9	1	3.5	Y	0.02	3.7	327	Y	14.5mo.		X
T3	53	M	HCV	27.6	1	3.5	Y	N/A	5	263	N			X
T4	54	M	HCV	67	3	2.9, 1.6, 1	Y	N/A	3	153	N			X
T5	58	M	HCV	4	1	7.6	N	0	7.6	82	N		X	
T6	59	M	HCV	4	2	3.1, 3.5	N	0.03	3.5	127	N		X	
T7	65	F	HBV	667	1	2.5	Y	0.33	2.5	167	N			X
T8	68	F	HCV	18	1	6	N	N/A	4.7	174	N		X	
T9	57	M	HCV	43	2	3.2, 1.8	N	N/A	3.2	141	N		X	
T10	48	M	HCV	40	4	3.5, 2.8, 2.5, 1.0	N	0.23	3.5	190	Y	6mo.	X	
T11	49	M	HCV	2000	1	3.5	Y	0.003	3.5	73	N			X
T12	53	M	HCV	189	3	2.4, 0.6, 0.3	Y	N/A	2.4	377	N			X
T13	54	M	HCV	8	5	2.1, 1.5, 1.4, 1, .7	N	0	4	241	N		X	
T14	58	M	HCV	188	1	6.3	N	0.12	5.5	345	Y	13mo.	X	
T15	57	M	HCV	3	3	2.8, 1.9, 1.3	Y	N/A	2	263	Y	18.5mo.		X
T16	62	M	HBV	20	2	1.7, 1.6	Y	0	2	153	N			X

A = Downstage to achieve eligibility.

B = Bridge to transplant.

* = Days from HCC diagnosis to transplant.

Confluent coagulative necrosis was present in all post-TACE HCC samples but not in any of the non-TACE HCCs. TACE treated tumors displayed greater necrosis, more fibrosis and less viable cells than non TACE treated samples. There were no differences in cell morphology, state of differentiation or nuclear/cytoplasmic ratio between the viable treated and nontreated tumor cells.

### Immunohistochemical characteristics of HCC tumors

All the presumptive CSC biomarkers were detected in either membranes or cytoplasm of some tumor cells. They showed a variety of staining patterns, including differences in staining intensity and percentage of positive cells. Duplicate sections or different areas for each tumor showed a good level of homogeneity for both stained cell percentages and intensities. Figure 1 demonstrates examples of the immunostaining patterns observed as an example for each expression intensity category.

**Figure 2:**
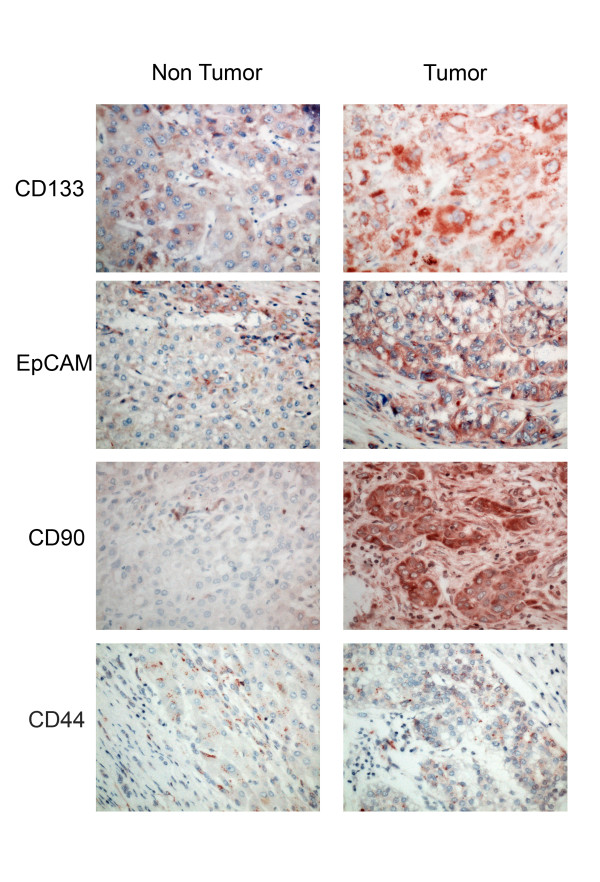
**Marker immunostaining in tumor and uninvolved liver.** Sections were immunostained as described and comparison samples are shown of HCC and surrounding non-tumor liver from the same specimens for CD133, EpCAM, CD90 and CD44.

The expression score of each of the markers was significantly higher in tumor cells than in adjacent liver tissue (Table 4, Figure 2). The expression of CD133 and EpCAM was significantly higher in the post-TACE group than in the non-TACE group (Table 4). There was no significant difference in CD44 and CD90 staining intensity in the TACE and non-TACE groups.

**Table 4:** Comparison of immunhistochemical staining score between HCC and adjacent liver tissues in TACE-treated and untreated tumors

	**HCC tissues (n-39)**	**Adjacent tissues (n = 39)**	**P**	**TACE (n = 16)**	**No-TACE (n = 23)**	**P**
**CD90**	1.3 ± 0.9	0.6 ± 0.7	0.002	1.69 ± 0.87	1.17 ±0.83	0.856
**CD44**	2.0 ± 1.0	1.5 ± 0.8	0.013	2.06 ± 0.85	1.95 ± 165	0.743
**CD133**	1.6 ± 1.2	1.2 ± 0.9	0.042	2.25 ± 1.00	1.17 ± 1.07	0.003
**EpCAM**	1.5 ± 1.1	0.6 ± 0.6	<0.001	2.00 ± 1.03	1.17 ± 0.89	0.016

As CD133 and EpCAM were the markers that associated with post-TACE status, we wanted to determine if variable expression of these markers predicted tumor characteristics. Post-TACE tumors were categorized into subgroups with either low (scores 0 and 1) or high (scores 2 and 3) expression of either CD133 or EpCAM. Clinical-pathological characteristics of patients with tumors in these categories are summarized in Table 5. There were no differences in gender, time interval between TACE and transplantation, number of TACE procedures performed, age, serum AFP, tumor size, or TNM stage in any of the groups. Tumor differentiation, however, was different between the EpCAM high and EpCAM low groups (p = 0.024). Univariate analysis showed that high EpCAM status was significantly associated with more undifferentiated tumor histology and having undergone TACE (Table 6). Multivariate analysis showed that the relative risk of having high EpCAM staining was independently influenced only by TACE status (relative risk 5.88 for TACE vs. no TACE, Table 7).

**Table 5:** Impact of marker status on clinical and pathological tumor features in TACE treated HCC

	**EpCAM**			**CD133**		
	**Low (n = 6)**	**High (n = 10)**	***P***	**Low (n = 4)**	**High (n = 12)**	***P***
**Age (year)**	58(53–65)	56(48–68)	0.236	52(48–57)	58(49–68)	0.070
**Gender (F/M)**	1/5	1/9	0.696	0/4	2/12	0.383
**AFP (ng/ml)**	1333(4–7254)	256(3–2000)	0.289	297(8–43)	870(3–7254)	0.177
**Interval between**	189(82–345)	224(73–337)	0.479	260(188–327)	194(73–377)	0.217
**TACE and Tx (days)**						
**More than one TACE**	1/6	3/10	0.639	2/4	2/12	0.329
**Tumor Size (mm)**	45(25–76)	34(20–55)	0.181	36(32–40)	39(20–76)	0.744
**Tumor differentiation**			0.024			0.834
Well	0	1		0	1	
Moderately	6	3		3	8	
Poor	0	6		1	3	
**TNM Stages**			0.302			0.248
I	4	4		1	7	
II	2	6		3	5	

**Table 6:** Univariate analyses of clinical and tumor factors associated with EpCAM/CD133 in 39 HCC patients

**Variables**		**Epcam**			**CD133**	
	**P value**	**OR**	**95% CI**	**P value**	**OR**	**95% CI**
**Age (≥55/<55)**	0.719	1.27	0.34-4.73	0.726	1.25	0.39-4.36
**Gender (Female/male)**	0.656	1.50	0.25-9.00	0.101	4.17	0.76-23.07
**AFP (ng/ml) (≥20/<20)**	0.066	3.75	0.92-15.34	1.000	1.00	0.29-3.48
**Tumor number (≥2/1)**	0.206	2.37	0.62-9.03	0.162	0.43	0.13-1.41
**Tumor size,cm (≥3/<3)**	0.547	0.67	0.18-2.49	0.292	1.89	0.58-10.93
**TNM stage**	0.206	2.37	0.62-9.25	0.068	0.30	0.08-1.10
**Differentiation**	0.033	5.36	1.15-25.06	0.154	2.4	0.72-7.97
**(well/moderate/poor)**						
**TACE treatment/not**	0.012	6.43	1.57-27.45	0.070	3.47	0.90-13.31

AFP,α-fetoprotein; TACE, Transarterial chemoembolization.

**Table 7:** Multivariate analyses of clinical and tumor factors associated with Epcam in 39 HCC patients

**Variable**	**P value**	**OR**	**95%CI**
**Differentiation (well/moderate/poor)**	0.056	5.01	0.96-26.21
**TACE Treatment/not**	0.025	5.88	1.25-27.80

### Impact of CSC markers on tumor recurrence

**Figure 3:**
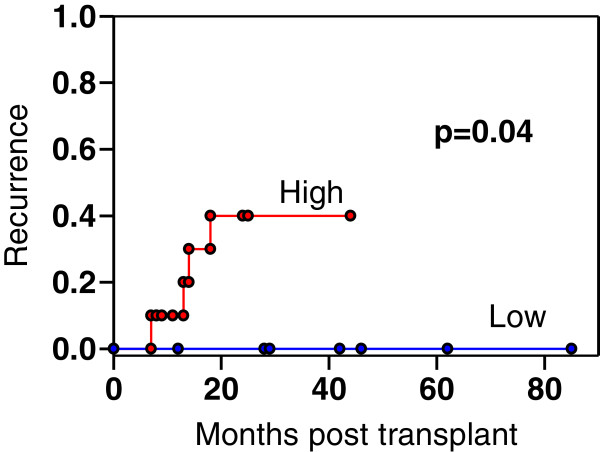
**Proportion of post-TACE patients experiencing tumor recurrence after transplantation.** The 16 patients were stratified by EpCAM staining intensity into high and low staining groups. Proportion of patients experiencing radiologically documented tumor recurrence is shown. For the high EpCAM staining patients, 4/10 recurred as compared to 0/6 low staining patients (P = 0.04).

We next examined whether EpCAM or CD133 staining intensity correlated with tumor recurrence. All patients included in this study were followed for at least two years post transplant. Four of the sixteen post-TACE patients had tumor recurrences. There was no effect of CD133 staining on recurrence. Two of the recurrences were in the 12 patients with CD133 high tumors and 2 were in the 4 patients with CD133 low tumors. In contrast, all 4 recurrences were in the 10 patients with EpCAM high tumors. None occurred in the 6 patients with EpCAM low tumors (median follow-up period, 33 months). Probability of recurrence in these groups is presented in Figure 3. Tumor recurrence rates at post-transplantation were 40 % (4/10) in EpCAM high and 0% in EpCAM low post-TACE patients (Figure 3, p = 0.040). In the 23 non-TACE treated patients, there was only one recurrence so the impact of marker staining could not be assessed.

## Discussion

The recognition that adherence to size and invasion based criteria such as the Milan criteria greatly reduces post-transplant tumor recurrence [5] has made it is possible to improve survival outcomes for large numbers of cirrhotic patients with relatively small HCCs. The development of effective therapies, such as TACE, that shrink tumor size has allowed a larger number of patients to undergo transplantation [9]. However, with increasing understanding that factors other than tumor size may predict tumor phenotype, there is a need to revisit the optimal criteria for predicting post-transplant recurrence. The recent findings that certain progenitor cell markers portend poor prognosis of HCC [20] promoted us to investigate the role of these in post-transplant tumor recurrence.

Hepatocellular carcinoma has a number of characteristics that are compatible with the existence of a circulating cancer stem cell (CSC) population [22,23]. The tumors are heterogeneous, frequently demonstrate intrahepatic recurrence after liver transplantation in spite of the absence of known metastatic disease, and different subpopulations of cells derived from HCCs have dramatically different abilities to form tumors in immunodeficient mice [15,16]. While phenotypic identification of these cells within tumors has been controversial, evidence exists supporting the importance of CD133, CD90, CD13, EpCAM and CD44 as potential markers to identify this cell population [14-19,24]. These observations have led to the widespread belief that CSCs are an important part of HCC biology. In spite of this consensus, identification of CSCs has been difficult due to lack of specificity of markers and limitations of model systems.

We reasoned that true HCC CSCs might be enriched in tumor cells that survived doxorubicin-based chemoembolization (TACE) and took advantage of the fact that some, but not all patients at our center undergoing liver transplantation for HCC underwent TACE prior to transplant. We thus stained HCC specimens and adjacent liver tissue for CD133, CD44, CD90, and EpCAM. Each of these has been previously reported to be associated with HCC CSCs. CD133 was used to identify CSCs in several types of tumors including HCC [19]. CD44, a stem cell biomarker, has been used as a target to attempt to eradicate CSCs in HCC [17,24] and has also been linked to invasion and metastatic potential [25,26]. EpCAM, a progenitor cell marker, was previously correlated with poor prognosis of HCC [15,27], and CD90 was reported as a key marker for the selection of CSCs [16].

The results showed that all of these markers were elevated in liver tumor tissues compared to adjacent non-tumor liver, but there were considerable differences between individual tumors. This suggests that the expression patterns of various stem cell markers in tumor initiating cells with stem/progenitor cell features may be different in each HCC, possibly due to the heterogeneity of activated signaling pathways in normal stem/progenitor cells where these tumor initiating cells may originate. Expression of CD133 and EpCAM were significantly elevated in post-TACE tumors. This finding is similar to the recent observation that HCC tumors recurring post TACE frequently had a morphology suggestive of a mixed hepatocholangiocellular phenotype that stained positive for stem cell markers [11]. The greater presence of CD133 and EpCAM positive cells in the post-TACE samples could result either because this cell population selectively survived treatment, or because recurrent tumor growth resulted in a dedifferentiation process that generated positive cells. The present study is not able to distinguish between these possibilities. In addition, since this was a retrospective study, we did not have samples from these tumors before TACE, and the decision to perform TACE was made on clinical grounds. It is therefore possible that the TACE patients had larger or more aggressive tumors. The TACE-treated tumor did tend to be larger than the non-TACE tumors but we were not able to detect any differences in pre-op tumor growth rates or TMN score between the two groups (Table 1).

Because of the possible difference between TACE-treated and non-TACE treated tumors we analyzed recurrence data separately for these two groups. We observed that the intensity of EpCAM, but not CD133 staining was a strong predictor of tumor recurrence in patients who were transplanted after TACE treatment. This was not true for the other markers studied. It supports prior studies that have identified EpCAM as a marker that best identified HCC-derived cells with tumor forming potential [15,28].

The results obtained in this study show a significant relationship between tumor recurrence rates and pretransplantation EpCAM expression in patients who underwent pre-transplant TACE. Recurrence only occurred in 1 of 23 patients without TACE as compared to 4 of 16 patients who had had prior TACE and thus the infrequency of the event in the non-TACE group made it impossible to assess correlations.

## Conclusion

Improvement of the ability to predict post-transplant HCC recurrence would allow pre-transplant characterization to more efficiently allocate organs, possibly increasing eligibility for patients with low recurrence risk and reducing the overall recurrence rate. While the understanding of histochemical markers in this study and others is an important step, it cannot be immediately translated into clinical practice due to the fact that HCC is not routinely biopsied, and heterogeneity within tumors makes in doubtful that a needle biopsy would be sufficient to make the required distinctions. Nonetheless, a better understanding of the biology associated with recurrence might allow better diagnostic studies, such as serum markers, to have impact in the future.

If tumor growth and metastasis are indeed driven by CSCs, this can explain why current chemotherapies, developed largely against the bulk tumor mass, are able to shrink the primary tumor, but are unable to provide a lasting cure for the disease. It is likely that these residual CSCs are able to survive, perhaps in circulation or other extrahepatic sites, and result in tumor relapse after transplantation. Better identification of CSC from tumor tissue may therefore be useful to target future treatments. Our study provides evidence EpCAM may prove to be a useful marker for HCC recurrence after liver transplantation and might be helpful in getting a better prediction of recurrence in patients who have been “downstaged” to fit into the Milan criteria. It will be important to replicate these results in larger cohort groups and determine whether other stem cell markers can also predict recurrence.

## Competing interests

The authors declare that they have no competing interests.

## Authors’ contributions

ZZ and SW conceived the design of the experiments, ZZ, JR, JZ, and MO performed immunostaining and/or histological analysis, ZZ, SW, BA, SK and TS collected and validated clinical data. BB oversaw human studies regulatory compliance, ZZ, MO, JC and SW analyzed the data, ZZ, JC and SW wrote the paper. All authors read and approved the final manuscript.

## Pre-publication history

The pre-publication history for this paper can be accessed here:

http://www.biomedcentral.com/1471-2407/12/584/prepub
